# Macrophage Membrane-Camouflaged shRNA and Doxorubicin: A pH-Dependent Release System for Melanoma Chemo-Immunotherapy

**DOI:** 10.34133/2022/9768687

**Published:** 2022-02-08

**Authors:** Chengli Yang, Yang Ming, Kai Zhou, Ying Hao, Danrong Hu, Bingyang Chu, Xinlong He, Yun Yang, Zhiyong Qian

**Affiliations:** ^1^State Key Laboratory of Biotherapy and Cancer Center, West China Hospital, Sichuan University, and Collaborative Innovation Center of Biotherapy, Chengdu, Sichuan 610041, China; ^2^Department of Pharmacy, The Affiliated Hospital of Guizhou Medical University, Guiyang, Guizhou 550000, China

## Abstract

Improving the efficacy of melanoma treatment remains an important global challenge. Here, we combined chemotherapy with protein tyrosine phosphatase nonreceptor type 2(Ptpn2) based immunotherapy in an effort to address this challenge. Short-hairpin RNA (shRNA) targeting Ptpn2 was coencapsulated with doxorubicin (DOX) in the cell membrane of M1 macrophages (M1HD@RPR). The prepared nanoparticles (NPs) were effectively phagocytosed by B16F10 cells and M1 macrophages, but not by M0 macrophages. Hence, NP evasion from the reticuloendothelial system (RES) was improved and NP enrichment in tumor sites increased. M1HD@RPR can directly kill tumor cells and stimulate immunogenic cell death (ICD) by DOX and downregulate Ptpn2. It can promote M1 macrophage polarization and dendritic cell maturation and increase the proportion of CD8^+^ T cells. M1HD@RPR killed and inhibited the growth of primary melanoma and lung metastatic tumor cells without harming the surrounding tissue. These findings establish M1HD@RPR as a safe multifunctional nanoparticle capable of effectively combining chemotherapy and gene immunotherapies against melanoma.

## 1. Introduction

Malignant melanoma is an aggressive tumor that readily develops resistance to treatment [1]. Early melanoma can be cured by surgical resection and has a long survival rate (SR; 5-year SR > 90%). However, once metastasized, the 5-year SR drops markedly to about 5% [2, 3]. Chemotherapy, including monotherapy or combination chemotherapy, has become the primary treatment modality for patients with advance stages of cancer and metastasis. Nevertheless, with standard regimens of dacarbazine and temozolomide, the median survival time is less than one year [4–8]. Therefore, new therapeutic approaches for melanoma are urgently required.

Melanoma is highly immunogenic and sensitive to immunotherapy, making immunotherapy a viable therapeutic option that has become a hot topic for research into personalized therapy [9]. Currently, the primary strategies for melanoma immunotherapy include systemic cytokine therapy [10, 11], immune-checkpoint inhibitor therapy [12, 13], and adoptive cell immunotherapy [14, 15]. However, the occurrence and development of melanoma result from multigene variation and multifactor interactions, making it difficult for a single immunotherapy to achieve the desired effect in every case [16, 17]. Consequently, combined immunotherapy has emerged as a potential way to tackle a broad spectrum of melanoma types. For example, immunotherapies such as interferon-alpha (IFN-*α*) or interleukin-2 (IL-2) have been used in combination with dacarbazine in the past [18]. Subsequently, ipilimumab, the Cytotoxic T-Lymphocyte Antigen 4 (CTLA-4) blocking monoclonal antibody and BRAF^V600E^ kinase inhibitor vemurafenib have been approved by the Food and Drug Administration (FDA). In combination, these therapies can somewhat improve outcomes and 5-year SR but are often also accompanied by gastrointestinal, endocrine, and immune overreaction and other adverse reactions [19].

Hence, the need remains to improve the therapeutic effect while at the same time reducing adverse reactions. In this regard, targeted therapies show numerous advantages. For example, the protein tyrosine phosphatase nonreceptor type 2 (Ptpn2) is highly expressed in melanoma and can reduce anti-tumor immunity by reducing the frequency of progenitor T cells maturing to killer T cells and interfering with killer T cells responding to IFN-*γ* signals. Thus, reducing the expression of Ptpn2 may provide a new scheme for the target immunotherapy of melanoma [20–24]. In addition, recent studies have reported that many chemotherapeutic drugs, including anthraquinones (e.g., doxorubicin (DOX) and mitoxantrone), can stimulate the immune response through the mechanism of immunogenic cell death (ICD) [25–27]. Therefore, such a combination of immunotherapy and chemotherapy can enhance the ICD mechanism of tumor cells by inducing and enhancing antigen presentation, thereby activating T cells and enhancing the immune response [28, 29].

Here, immunotherapy based on the Ptpn2 gene was combined with DOX chemotherapy for the treatment of malignant melanoma. We developed novel nanodelivery systems in order to realize its application *in vivo*. Inspired by the phospholipid bilayers of cell membranes including those of erythrocytes and tumors, many functional liposome particles have been designed for the purpose of biotherapy [30, 31]. Nanosystems enshrouded by macrophage membranes can escape from the RES [32]. Polarized M1 macrophage membranes were used to further improve the targeting effect of our NPs [33].

**Figure 1:**
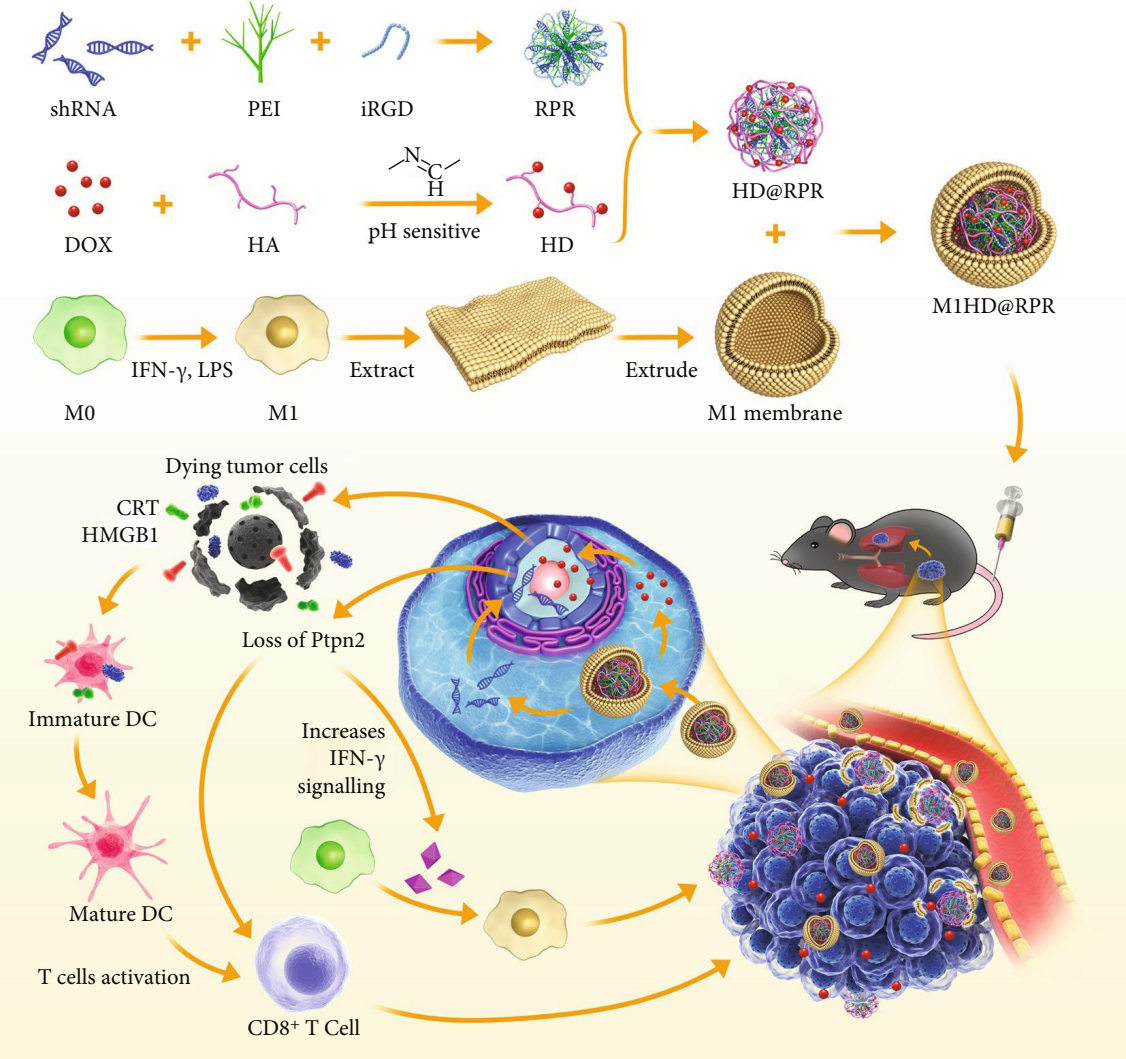
Schematic illustration of the M1HD@RPR construction and its application in combined cancer therapy.

We encapsulated pH-sensitive DOX and nuclear targeting shRNA-Ptpn2 with M1 macrophage membrane to achieve concurrent targeted delivery of an immune-related gene and chemotherapeutic. First, the pH-sensitive modified DOX was synthesized via a simple Schiff base reaction between DOX and partially oxidized hyaluronic acid (OHA). Meanwhile, to increase the transfection efficiency of the shRNA-Ptpn2 plasmid, tumor-homing peptides iRGD and branched polyethylenimine (PEI) were used to condense shRNA-Ptpn2 and form a complex (shRNA-PEI-iRGD, RPR). The RPR surface was coated with HA-DOX (HD) via electrostatic adsorption to form HD@RPR. Finally, the polarized M1 membranes were used to camouflage HD@RPR. The shRNA-Ptpn2 system combined with DOX was then administered as immuno-combined chemotherapy to a mouse model of melanoma via nanoparticles, and the therapeutic efficacy was assessed (Figure 1).

## 2. Results and Discussion

### 2.1. Construction and Characterization of M1HD@RPR

Exploiting pH sensitivity in tumor sites is an effective strategy for targeted delivery of antitumor drugs [34–36]. Here, we created a pH-sensitive DOX by Schiff base reaction between DOX and HA (HA-DOX). The structures of OHA and HA-DOX were confirmed using ^1^H-NMR and FTIR.

The ^1^H-NMR results showed that compared with HA, OHA had an aldehyde proton peak at 8.3 PPM and a resonant hemiacetal proton peak at 5.0-5.25 ppm, indicating that HA had been successfully oxidized to OHA. Compared with free OHA and DOX, in HA-DOX, the aromatic ring proton peak at 6.85-7.25 ppm weakened and the amino proton peak at 7.85 ppm disappeared, while the aldehyde group proton peak weakened at 8.3 ppm, showing that HA-DOX had been successfully synthesized. FTIR spectra showed that compared with HA, a typical C=O stretching band of the aldehyde group appeared at 1731 cm^–1^ for OHA, while C(O)-H at 2750 cm^–1^ coexisted with the C-H absorption peak of HA itself. Compared with the FTIR of OHA, the HA-DOX spectrum showed a stretching band of the DOX aromatic ring at 1604 cm^–1^. Therefore, both FTIR and ^1^H-NMR indicated that HA-DOX was successfully synthesized (Figures S1 and S2). DOX content was determined by UV-Vis, and the results showed that the yield of the HA-DOX synthesis reaction was approximately 94% while the final DOX grafting rate on HA was approximately 12%.

**Figure 2:**
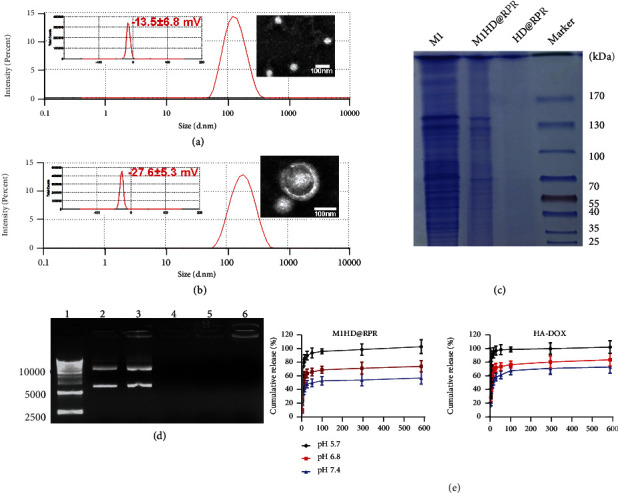
Characterization of nanoparticles. (a) and (b) Size distribution, zeta potential (inset graph), and morphology of HD@RPR and M1HD@RPR (inset image). (c) Protein profiles in the M1, HD@RPR, and M1HD@RPR determined via SDS-PAGE assay. (d) DNA fragment migration by agarose gel electrophoresis. Lane 1, DNA ladder; lane 2, shRNA-Ptpn2 plasmid; lane 3-6: RR, RPR, HD@RPR, M1HD@RPR. (e) DOX-release profiles in the presence different pH values.

The particle size, zeta potential, and transmission electron microscopy (TEM) results of HD@RPR are shown in Figures 2(a) and 2(b). The self-assembled HD@RPR was a relatively uniform composite with a particle size of 123.6 ± 14.4 nm and an average surface charge of −13.5 ± 6.8 mV. Compared with HD@RPR, the particle size of M1HD@RPR markedly increased, reaching 155.2 ± 17.7 nm, and the surface charge was −27.6 ± 5.3 mV. TEM results showed that M1HD@RPR had a core-shell structure.

Thereafter, protein profiles were detected in M1 macrophage membranes and M1HD@RPR using SDS-PAGE. In addition, most of the proteins in the M1 macrophage membranes of M1HD@RPR were retained, while no protein signal was detected in HD@RPR nanoparticles (Figure 2(c)).

An agarose gel condensation assay was performed to verify the DNA protective effect of RR, RPR, HD@RPR, and M1HD@RPR on shRNA-Ptpn2. As shown in Figure 2(d), except for the positive charge of iRGD, which was too small to condense the shRNA-Ptpn2 plasmid, all other components effectively blocked shRNA-Ptpn2 in the gel pore.

### 2.2. pH-Sensitive Release of DOX

The mildly acidic environments in the tumor (approximately 6.5), endosomes (pH 5-6), and lysosomes (pH 4-5) can trigger on-demand drug release [34]. Accordingly, the pH-sensitive release of DOX, monitored in PBS at different pH values of 5.7, 6.8, and 7.4, was investigated. The relative release rate of HA-DOX and M1HD@RPR in different pH environments was pH 5.7 > pH 6.8 > pH 7.4. At pH 5.7, almost 100% of DOX was released after 24 h. While at pH 7.4, the cumulative release was <80% even after 600 hours. This result implied that DOX release would be reduced under physiological pH conditions, thus, reducing DOX cardiotoxicity. Notably, under different pH conditions, the release rate of DOX from free HA-DOX was higher than from M1HD@RPR, suggesting that the M1HD@RPR-wrapped HA-DOX could specifically release DOX to the tumor site (Figure 2(e)).

### 2.3. Cellular Uptake and Permeability of Nanoparticles

**Figure 3:**
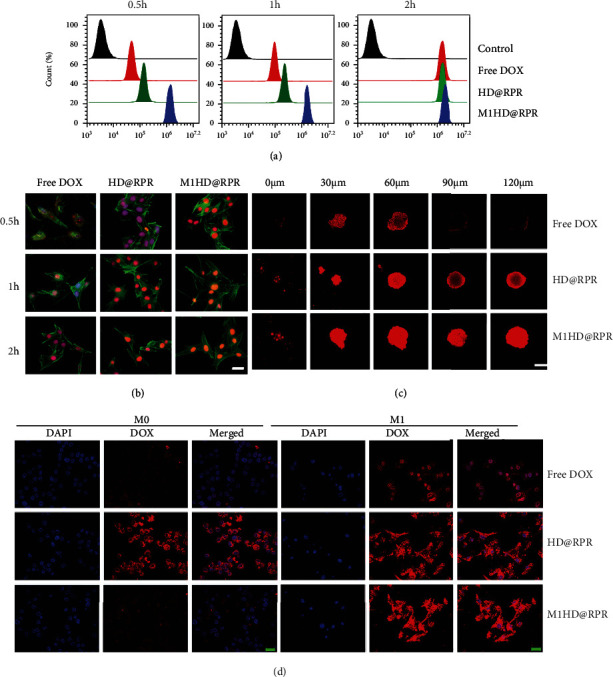
Cellular behavioral studies. (a) Flow cytometry analysis of B16F10 cellular uptake of Free DOX, HD@RPR, M1HD@RPR at 0.5 h, 1 h, and 2 h. (b) Distribution of DOX uptake investigated by cytoskeleton labeled with FITC-phalloidin, (scale bar: 20 *μ*m). (c) Study on penetration ability of nanoparticles into 3D tumor spheroids. (d) Uptake of free DOX, HD@RPR, and M1HD@RPR by macrophages (scale bar: 20 *μ*m).

Cellular uptake of Free DOX, HD@RPR, and M1HD@RPR exhibited a time-dependent effect within 2 h, with the fluorescence intensity of DOX significantly increasing within cells after 2 h compared with 0.5 h (Figure 3(a)). Moreover, at 0.5 h, the uptake efficiency of M1HD@RPR was significantly higher than that of Free DOX; however, this difference gradually decreased with increasing time as the uptake of DOX by cells was saturated. In contrast, no significant difference was observed in the release rate of HD@RPR and M1HD@RPR.

Additionally, following staining of the cytoskeleton with phallacidin DOX was found to be retained in the cytoplasm at 0.5 h in the free DOX group, entering the nucleus only gradually with increased time. Meanwhile, in the HD@RPR and M1HD@RPR groups, DOX entered the nucleus within the first 0.5 h. This effect was caused by the pH-sensitive modification of DOX causing its rapid release in the acidic endosomes and lysosomes (Figure 3(b)).

The ability to penetrate and infiltrate the interior of a solid tumor is a key characteristic of effective drug/gene carriers. To determine whether M1HD@RPR nanoparticles can penetrate tumors *in vitro*, 3D tumor spheres that simulated tumors and tumor microenvironments were created. When the tumor depth was 120 *μ*m, only weak fluorescence was observed at the edge of the tumor sphere in the Free DOX group. Whereas strong red fluorescence was observed in the center of the 3D tumor sphere treated with NPs, indicating that the cell uptake was significantly enhanced via encapsulation of the NPs. Moreover, the uptake was further enhanced following addition of the M1 membrane envelope, possibly due to membrane fusion (Figure 3(c)).

Lastly, the uptake of Free DOX, HD@RPR, and M1HD@RPR nanoparticles by M0 and M1 macrophages was investigated. The uptake of HD@RPR by M0 cells was significantly higher than that of free DOX, possibly due to nanoparticle endocytosis enhancing DOX uptake (Figure 3(d)). Interestingly, the encapsulation with M1 cell membranes significantly reduced the uptake efficiency of HD@RPR by M0, which may be caused by different cellular uptake mechanisms. However, the results indicated that the encapsulation of M1 cell membranes reduced the clearance rate of M0 macrophages, thus, extending the circulation time of nanoparticles *in vivo*. In addition, the results of the M1 macrophage uptake study showed that, in general, following polarization of macrophages into M1, the overall phagocytosis effect was significantly enhanced, the uptake efficiency of HD@RPR and M1HD@RPR was increased, and the encapsulation of M1 cell membranes did not impact M1 macrophage uptake.

### 2.4. Efficient Silencing of Ptpn2 In Vitro

**Figure 4:**
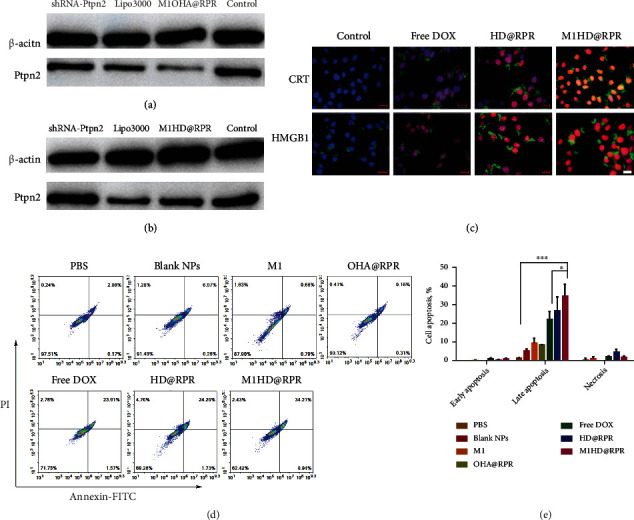
Cellular pharmacodynamics. (a) and (b) Ptpn2 protein expression in B16F10 cells determined by western blotting after different treatments.(c) Immunofluorescence detection of CRT and HMGB1 expressed on B16F10 cell (scale bar: 20 *μ*m).(d) and (e) Apoptosis of B16F10 cells treated with different formulations for 12 h.

The expression of Ptpn2 protein after shRNA-Ptpn2 transfection was detected by Western blotting. First, the silencing effect of DOX-free nanoparticles on Ptpn2 protein in B16F10 cells was investigated; Ptpn2 protein expression was significantly inhibited after B16F10 was treated with M1OHA@RPR nanoparticles (M1HD@RPR nanoparticles without DOX) while the Lipofectamine 3000 (abbreviated as Lipo3000) group showed no significant difference (Figure 4(a)). Thus, the nanoparticles achieved efficient intracellular delivery of shRNA-Ptpn2 and silenced Ptpn2, which is required for effective immunotherapy *in vivo*.

The effect of M1HD@RPR nanoparticles on Ptpn2 silencing was further investigated, the results in Figure 4(b) show that although most cells underwent apoptosis due to DOX, and they maintained a high gene transfection efficiency and, thus, exhibited a moderate gene silencing effect.

### 2.5. Cellular ICD Effect Assay

ICD, a form of tumor-cell apoptosis induced by various chemotherapeutic agents including anthracyclines, can lead to CRT exposure and HMGB1 release, while enhancing the recognition and phagocytosis of dying cancer cells. In this study, the expression of CRT and HMGB1 was detected by immunofluorescence of Free DOX, HD@RPR, and M1HD@RPR, with PBS used as the control (Figure 4(c)). The abundance of CRT and HMGB1 (green fluorescence channel) in HD@RPR and M1HD@RPR nanoparticles was significantly higher than that in the free DOX group, and the red fluorescence of DOX was also significantly enhanced compared with free DOX. Hence, the nanoparticle group enhanced the uptake of DOX and thus enhanced the ICD effect of DOX.

### 2.6. Nanoparticles Can Effectively Kill Tumor Cells In Vitro

To investigate the effect of nanoparticles on tumor cell apoptosis and cell killing, B16F10 cells were simultaneously stained with Annexin V-FITC/PI and Calcein-AM/PI. In the Blank NPs group, 0.26% of the cells showed early apoptosis and 6.97% of the cells showed necrosis or late apoptosis; while in the single M1 cell membranes treatment group (abbreviated as M1 group), 0.79% and 9.68% showed early and late apoptosis, respectively, indicating that M1 cell membranes and blank nanomaterials exhibited negligible levels of toxicity to tumor cells *in vitro* (Figures 4(d) and 4(e)). Moreover, the apoptosis rate in the OHA@RPR group (nanoparticles with shRNA-Ptpn2 alone) was similar to that of the Blank NPs group (Blank nanoparticles group), indicating that the downregulation of *Ptpn2* in the absence of a tumor microenvironment had almost no effect on inducing cell killing. By contrast, in the combined gene therapy groups, DOX uptake efficiency was enhanced due to nanoparticle delivery, and apoptosis was enhanced. After envelopment with M1 macrophage membranes, the apoptotic effect induced by NPs was further enhanced, reaching approximately 35%.

In the Calcein-AM/PI experiment, the toxicity of the PBS, Blank NPs, M1, and OHA@RPR groups was negligible, with only a small amount of PI staining detected, which corresponded with the results of the Annexin V-FITC/PI test. However, given the antitumor effect of DOX, the toxicity of the DOX containing group markedly increased (Figure S3).

### 2.7. BMDC Maturation Induction and M1 Macrophage Polarization

**Figure 5:**
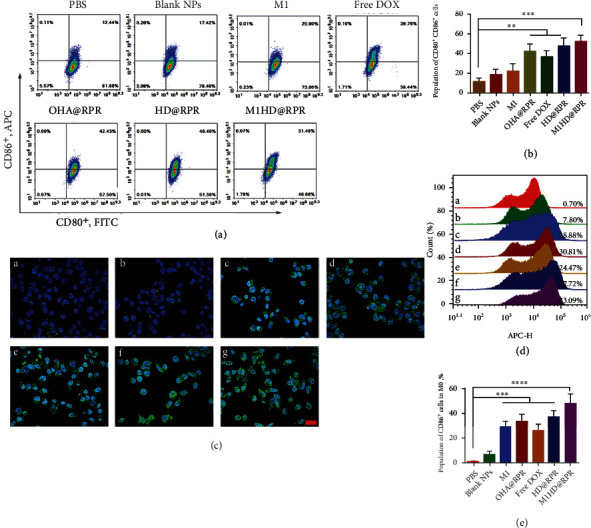
Study on cellular immunity mechanism. (a) and (b) Representative flow cytometry plots of CD80^+^ CD86^+^ in each group. (c) Immunofluorescence results of M1 macrophages induced by nanoparticles. a, PBS; b, Blank NPs; c, M1; d, OHA@RPR; e, Free DOX; f, HD@RPR; g, M1HD@RPR (scale: 20 *μ*m). (d) and (e) Flow cytometry results of M1 induced by nanoparticles. a, PBS; b, Blank NPs; c, M1; d, OHA@RPR; e, Free DOX; f, HD@RPR; g, M1HD@RPR.

Dendritic cells (DCs) are the strongest dedicated antigen-presenting cells [37, 38]. Thus, B16F10 cells were treated with each component; the supernatants from each culture were then used to treat BMDCs. The abundance of CD11c, CD80, and CD86 was then assessed in cells via flow cytometry. The results showed that the proportion of CD11c^+^CD80^+^ cells in the M1 group was significantly higher than those in the PBS group, suggesting that the uptake of M1 by B16F10 cells could enhance the release of immune-related factors, thereby promoting the maturation of DCs. Mainly due to the uptake of DOX, shRNA-Ptpn2, and M1 into B16F10 cells, the proportion of CD11c^+^CD80^+^ in the M1HD@RPR group was significantly higher than the control. Accordingly, the release of inflammatory factors was enhanced leading to the maturation of DCs (Figure S4). In addition, as CD86 and CD80 are costimulatory molecules that activate T cells on the surface of DCs, they affect the numbers of CD80^+^CD86^+^ (Figures 5(a) and 5(b)). Compared with the control group, the proportion of CD80^+^CD86^+^ in M1HD@RPR group was significantly increased, indicating that the nanoparticles had a very high maturation-induction effect on DCs, thus, promoting antigen presentation and enhancing immunity.

Moreover, M1 macrophages can kill tumor cells and inhibit the formation of blood and lymphatic vessels of tumor cells [39, 40]. Many studies have shown that when macrophages transition from M0 to M1, the expression of CD86 increases. Hence, a cellular immunofluorescence assay was used to detect the expression of CD86 markers to visually observe the induction of M1 macrophages and the morphological changes of M0 to M1 cells. The green fluorescence of the PBS and Blank NPs group was barely visible, while that of the M1 and OHA@RPR group was enhanced. However, most cells maintained the circular shape of M0, indicating that some M0 had gradually transformed to M1 (Figure 5(c)). In addition, in the MIHD@RPR group, approximately 50% of the cells had developed the irregular morphology of M1 macrophages, indicating that our nanoparticles were involved in the polarization of M0 to M1. Further quantitative flow cytometry results are shown in Figures 5(d) and 5(e).

The effect of each group on the polarization of M2 into M1 is shown in Figure S5. The combination of IL4 + IL10 was found to polarize a proportion of the M0 cells into M2 (the average M2 ratio of the IL4 + IL10 group was 23.63% while that of the control group was 3.62%). However, no significant difference was observed in M1 polarization (the average M1 ratio of IL4 + IL10 group was 5.42% while that of the control group was 3.18%). The proportion of M1 and M2 cells in the Free DOX, HD@RPR, and M1HD@RPR groups was significantly higher than that in the PBS group.The results showed that DOX group could significantly promote the polarization of M1 and M2 cells simultaneously. However, the M1/M2 ratio statistics showed that the M1 proportion of the HD@RPR and M1HD@RPR groups was significantly increased, thereby enhancing the immunotherapeutic effect.

### 2.8. Investigation of DOX Distribution and Tumor Accumulation In Vivo

**Figure 6:**
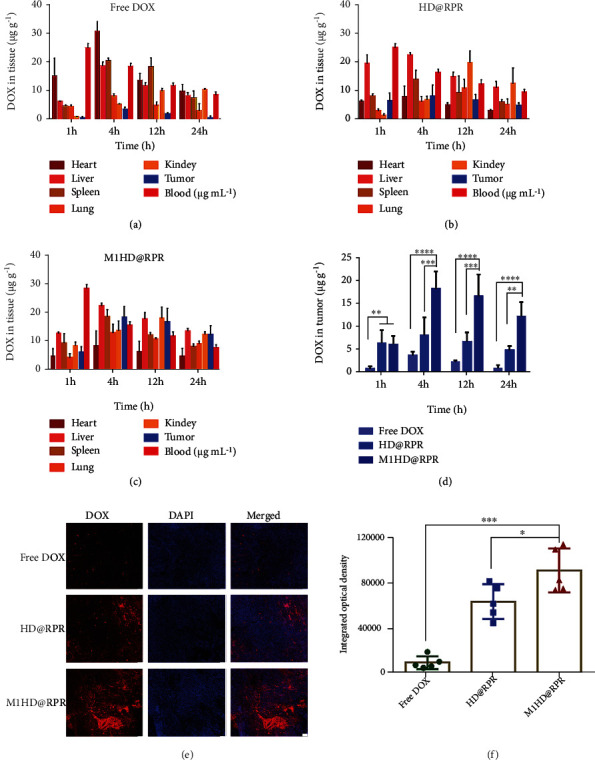
Tissue distribution of DOX after intravenous administration of (a) Free DOX (b) HD@RPR, and (c) M1HD@RPR. (d) DOX concentration in tumor tissues of each group. (e) and (f) CLSM images and its semiquantitative results of nanoparticles distribution in B16F10 tumor sections 24 h postinjection (scale bar: 200 *μ*m).

The content of DOX in different groups at each time point in blood and viscera of mice was measured by HPLC (Figures 6(a)–6(c)). In the HD@RPR group, due to the effect of the reticuloendothelial system, DOX was rapidly distributed in the liver and spleen and was less distributed in the heart. Moreover, the distribution of DOX in the tumor group was significantly increased compared with the Free DOX group due to the HA-DOX being sensitized to tumor microenvironment. After 24 h, the DOX content in the tumor site remained higher than that in the Free DOX group.

Due to the long circulation of cell membranes and weak scavenging effect of macrophages, the M1HD@RPR nanoparticles were distributed in the blood at 1 and 4 h. At 4 h, the maximum distribution of DOX was detected in the tumor site, and a high level of DOX was retained in the tumor at 24 h. Moreover, the DOX distribution for the M1HD@RPR group was significantly higher in the tumor site than that in the Free DOX and HD@RPR groups (Figure 6(d)), thus, proving that M1 membrane encapsulation could significantly improve the delivery of DOX to the tumor site.

To directly observe the distribution of Free DOX, HD@RPR, and M1HD@RPR in the tumor site 24 h after administration, paraffin-embedded sections were prepared and the distribution of DOX was observed by photography (Figures 6(e) and 6(f)).

### 2.9. Chemo-Immunotherapeutic Effect against Melanoma

**Figure 7:**
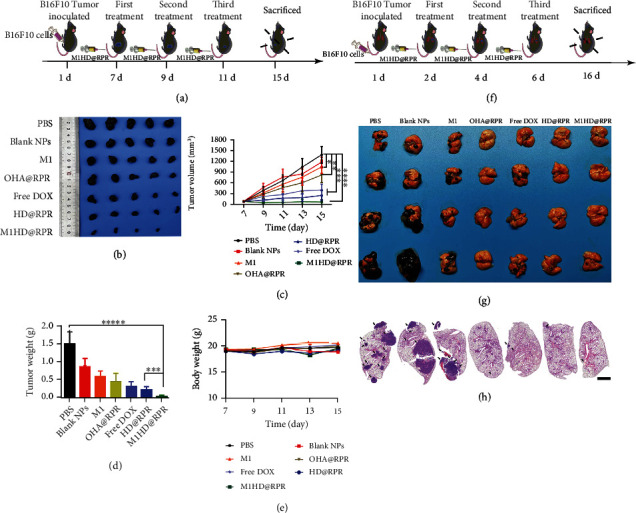
Chemo-immunotherapy effect for melanoma. (a) Scheme of treatment for primary tumor (*n* = 5). (b) Images of the tumors collected from various groups of mice after treatments. (c) Tumor volume in B16F10 tumor-bearing mice treated with different groups (*n* = 5). (d) Images of the tumors' weight collected from various groups of mice at the end of the treatments (*n* = 3). (e) Body weight of mice bearing B16F10 tumors after various treatments. (f) Scheme of treatment for pulmonary metastasis (*n* = 4). (g) and (h) Typical photographs and H&E staining of B16F10 metastatic foci for mice after different treatments, scale bar: 2000 *μ*m.

B16F10 tumor-bearing mice were established to evaluate the inhibitory effect of nanoparticles on primary tumors *in vivo* (Figure 7(a)). After the mice were euthanized, tumor tissues were removed, photographed, and weighed (Figures 7(b)–7(d)). The tumor volume in PBS group mice increased rapidly and grew to approximately 1500 mm^3^ on day 15 postinoculation. The growth curve of the Blank NPs group was similar to that of the PBS group. The final tumor volume of the M1 and OHA@RPR groups was significantly lower than that of the PBS group, indicating that the M1 cell membranes enhanced the maturation of DCs and generation of M1 macrophages; whereas the gene therapy enhanced the sensitivity of T cells to IFN-*γ*, to produce the immunotherapeutic effect. The tumor volume growth of the HD@RPR and M1HD@RPR groups was significantly slower than that of PBS, with some tumors found to disappear entirely, likely attributable to the sensitive release of DOX and the immunotherapeutic effect of the shRNA-Ptpn2 gene.

Furthermore, as can be seen from Figure 7(e), the body weight of mice in each group was not significantly reduced, thereby demonstrating the safety of the nanomaterials.

**Figure 8:**
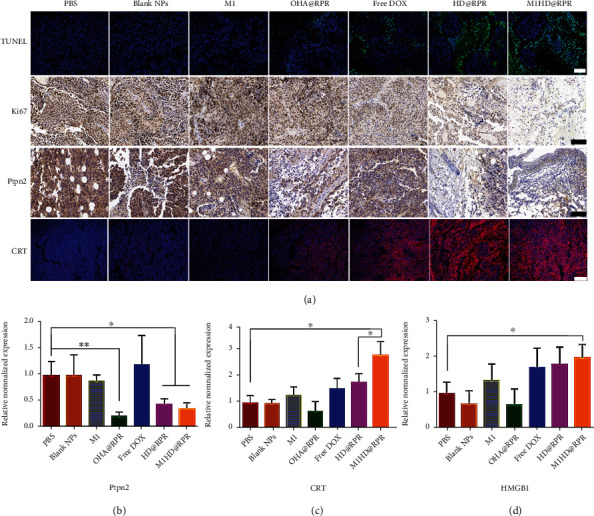
(a) Histological images of TUNEL, Ki67, Ptpn2, and CRT expression in tumor sections of different treatment groups (scale bar: 200 *μ*m). (b) to (d) mRNA expression of Ptpn2, CRT, and HMGB1 were detected by qPCR after treatment.

TUNEL, Ki67 assay, and H&E staining were performed (Figure 8(a) and S6) and revealed that the drug delivery system significantly enhanced the tumor tissue killing effect. In addition, H&E staining revealed a normal morphology of the main organs, with no abnormal inflammatory cell infiltration following treatment, thus, indicating that M1HD@RPR might significantly reduce cardiotoxicity [41]. Meanwhile, within the M1HD@RPR group, numerous cavities were observed in the tumor tissue and tumor nucleus pyknosis, suggesting that the nanoparticle treatment induced tumor cell apoptosis and tumor-specific tissue damage to inhibit tumor growth.

The growth of melanoma in the lungs of mice is shown in Figures 7(f)–7(h). Tumors of mice in the PBS and Blank NPs groups were almost completely covered with melanoma cells, and the lung tissues were seriously damaged, indicating that the melanoma was highly aggressive in the lungs. In addition, H&E results from the lungs of mice in each group revealed dense and clear tumor tissues occupying approximately 50% of the lung space. In the OHA@RPR, HD@RPR, and M1HD@RPR groups, very few melanoma cells were detected and the lungs remained intact. H&E results further revealed that the lung cells were intact, while the alveoli showed no expansion, and the structure was clear, suggesting negligible levels of lung metastatic tumor. Collectively, these results suggested that the administered nanoparticles had an excellent therapeutic effect on lung metastatic melanoma due to the activation of immunotherapy.

### 2.10. Ptpn2 Gene Silencing In Vivo

Messenger RNA (mRNA) levels in tumor tissues of mice in each group were analyzed after treatment (Figure 8(b)). The mRNA expression level of OHA@RPR was significantly lower than that in the PBS group, suggesting that shRNA-Ptpn2 significantly downregulated the expression of Ptpn2 protein in tumors, which was confirmed via IHC analysis (Figure 8(a)). Moreover, the mRNA expression level of the M1HD@RPR group was significantly lower than that of the PBS group but higher than that of the OHA@RPR group, which may have resulted from DOX killing a portion of tumor cells and reducing the transfection effect of the shRNA-Ptpn2 gene, thus, reducing the ability to downregulate Ptpn2 mRNA expression.

### 2.11. ICD Response Assessment In Vivo

As shown in Figures 8(c) and 8(d), the mRNA expression levels of CRT and HMGB1 in the M1HD@RPR group were significantly higher than those in the PBS group. These results indicate that nanoparticles increase the delivery of DOX, thus, enhancing its therapeutic ICD effect.

To observe the effect of ICD at the protein level, the abundance of CRT in tumor tissues was detected by immunofluorescence (Figure 8(a)). The CRT expression was barely observed in the PBS, Blank NPs, and OHA@RPR groups, while the associated red fluorescence was significantly increased in the Free DOX, HD@RPR, and M1HD@RPR groups, indicating that ICD enhances the expression of CRT in tumor tissue [42].

### 2.12. Tumor Microenvironment and Blood Immune-Related Cytometry Analysis

To investigate whether M1HD@RPR could stimulate the immunotherapeutic effect of tumor tissues by silencing Ptpn2, as well as the underlying ICD mechanism of DOX, we analyzed the proportion of CD8^+^ T cells in tumor tissues, the proportion of mature DCs in BMDCs, and the proportion of M1 macrophages.

**Figure 9:**
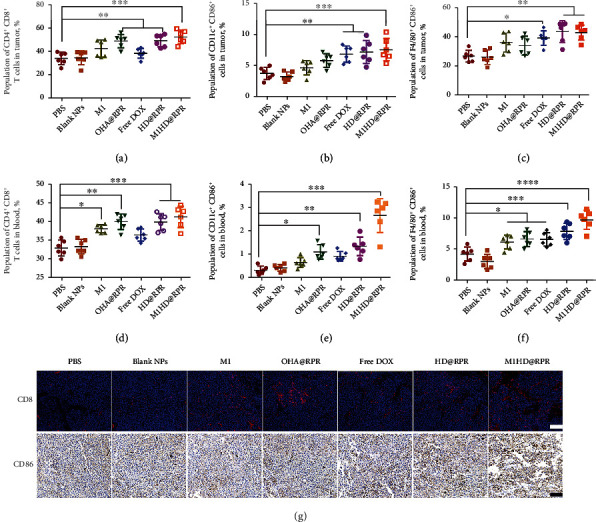
Study on immune mechanism. (a) to (c) The population of CD8^+^ T cells, CD11c^+^CD86^+^ cells, and F4/80^+^ CD86^+^ cells in the tumor. (d) to (f) The population of CD8^+^ T cells, CD11c^+^ CD86^+^ cells, and F4/80^+^ CD86^+^ cells in the blood.(g) Immunofluorescence and IHC analysis of CD3^+^ T cells (red) and CD86 in the tumor collected after the mice were subjected to different treatments (scale bar 200 *μ*m).

As shown in Figure S7, the proportion of CD8^+^ T cells in the shRNA-Ptpn2 treatment group (OHA@RPR, HD@RPR, and M1HD@RPR groups) was significantly higher than that in the PBS group. The representative flow cytometry analysis diagram of each group is shown in Figure 9(a). In addition, the proportion of CD8^+^ T cells in the DOX group (HD@RPR and M1HD@RPR groups) was higher than that in the gene therapy alone group (OHA@RPR group), showing that ICD can promote the exposure of CRT and HMGB1 protein on the cell surface. These results suggested that downregulation of Ptpn2 could increase the proportion of CD8^+^ T cells, thus, improving the immunotherapeutic effect against tumor tissue.

As shown in Figures 9(b) and S8, the average proportion of mature DCs (CD11c^+^ CD86^+^ cells) in the PBS group was significantly lower than that of the free DOX, HD@RPR, and M1HD@RPR groups, indicating that ICD immunopreparation of DOX can activate the antigen-presenting function to enhance the proportion of active DCs. Therefore, DOX not only directly promotes tumor cell apoptosis via chemotherapy but also activates the immune effect of the tumor microenvironment by increasing the number of dying tumor cells.

The proportion of M1 (F4/80^+^CD86^+^cells) macrophages in the F4/80 mononuclear macrophage population is presented in Figure 9(c) and S9. The average proportion of M1 macrophages in the PBS group was lower than that in the M1, OHA@RPR, Free DOX, HD@RPR, and M1HD@RPR treatment groups, consistent with the *in vitro* results. These results suggested that nanoparticle treatment promoted the release of IFN-*γ* and other cytokines, and that IFN-*γ* further promoted the transformation of M0 to M1 in the tumor microenvironment [43]. The proportion of M1 macrophages increased, which further stimulated the effect of tumor immunotherapy.

Similar results were found in the proportion of CD8^+^ T cells (Figures 9(d) and S10), mature DCs (Figures 9(e) and S11), and M1 macrophages (Figures 9(f) and S12) in the blood compared to those in tumors.

### 2.13. Immunofluorescence Detection of CD8^+^ T Cells and M1 Macrophages in Tumors

In addition, to intuitively investigate the changes of killer T lymphocytes in tumor tissue, paraffin-embedded sections of tumor tissues were used for immunofluorescence staining of CD8^+^ T cells. The results in Figure 9(g) showed that shRNA Ptpn2-mediated immunotherapy further improved the proliferation of CD8^+^ T cells. Moreover, compared with the gene therapy group (OHA@RPR), the proportion of CD8^+^ T cells was higher in the combination chemotherapy group (M1HD@RPR). This effect was caused by DOX promoting the ICD effect while inducing the chemotherapeutic killing of tumor cells; M1 membrane encapsulation enhances the targeting effect of M1HD@RPR tumor tissue.

The abundance of CD86 was measured in tumor tissues via IHC to analyze the effect of nanoparticles on inducing M1 macrophage polarization *in vivo* [44] (Figure 9(g)). There were few CD86^+^ cells in the control group while a significant increase occurred in the HD@RPR and M1HD@RPR groups, indicating that nanoparticles could play a role in tumor immunotherapy by increasing the proportion of M1 macrophages.

### 2.14. Detection of Immunorelated Cytokines in Tumor and Blood

Tumor tissues were extracted, and the expression levels of inflammatory factors were detected via qPCR assay. As shown in Figure S13, compared with the PBS group, the mRNA expression levels of IL-6, IFN-*γ*, and TNF-*α* were significantly increased in the MIHD@RPR group. Hence, downregulation of Ptpn2 and targeted delivery of DOX may enhance the sensitivity of CD8^+^ T cells, M1 macrophages polarization, and DC maturation, thus, promoting the related cytokine release to the tumor microenvironment.

A CBA protein assay was used to investigate the concentrations of IFN-*γ*, TNF-*α*, and IL-6 in serum (Figure S14). The results showed that the IFN-*γ*, IL-6, and TNF-*α* levels in the M1HD@RPR group were significantly higher than those in the PBS control group, indicating that the designed vector elicited an excellent immunotherapeutic effect.

### 2.15. Safety Evaluation

tComplete blood count (CBC) test and biochemical analyses were performed to investigate the safety of nanoparticles *in vivo*. Briefly, healthy mice were administered the same treatment regimen of nanoparticles as the tumor-bearing mice, and blood samples were collected 7 days following the end of treatment to observe changes in blood biochemistry and composition in mice. The results are shown in Figure S15. Compared with the PBS group, the CBC test did not differ in the other groups, indicating that the blank nanomaterials and drug-loaded nanoparticles had no evident toxicity while also exhibiting good biosecurity. In addition, there were no significant differences in AST, ALT, UREA, or CREA among all groups (*P* > 0.05). The AST and ALT in the free DOX group were higher than those in the PBS group due to the cardiotoxicity associated with DOX. However, compared with the PBS group, there was no significant difference between the Blank NPs group and the other nanotreatment groups (*P* > 0.05), indicating that the pH-sensitive modification of DOX effectively lowered its toxicity and the nanotreatment was safe *in vivo*.

## 3. Conclusion

We have constructed a pH-sensitive dual-modality targeted delivery system, M1HD@RPR, using electrostatic self-assembly to exert the effect of chemotherapy combined with immunotherapy on primary melanoma and metastatic tumors. shRNA-Ptpn2, which downregulated Ptpn2 expression, was designed and coencapsulated with pH-sensitive modified DOX wrapped in M1 macrophages. The prepared shell-core M1HD@RPR nanoparticles protected the shRNA-Ptpn2 and facilitated the pH-sensitive release of DOX. The encapsulation of the system by M1 membranes reduced the phagocytic vulnerability of HD@RPR to M0 macrophages, thus prolonging the circulation time *in vivo.* In addition, M1HD@RPR effectively downregulated Ptpn2 gene expression while producing a highly efficient melanoma-cell killing effect. Furthermore, our *in vitro* studies revealed that M1HD@RPR has an immunotherapeutic role by activating DCs, promoting the polarization of M0 to M1, and increasing the proportion of CD8^+^ T cells.

*In vivo* results showed that encapsulating M1HD@RPR in M1 membranes served to extend the circulation time *in vivo* and enhance fusion with tumor cells, thus, ensuring accumulation of DOX at the tumor site. Additionally, *in vivo* M1HD@RPR was found to significantly inhibit the growth of primary and lung metastatic melanoma. Tumor immune microenvironment analysis further revealed that downregulation of the Ptpn2 gene could enhance the activation of CD8^+^ T cells, polarization of M1 macrophages, and induction of DC maturation while promoting the release of cytokines, thus, eliciting an immunotherapeutic effect.

In this study, simple and readily prepared nanoparticles wrapped in macrophage membranes and loaded with genes and chemotherapy drugs were successfully prepared and found to elicit effective combined gene immunotherapy and chemotherapy. Specifically, these nanoparticles were successfully applied for the treatment of mouse melanoma in situ and lung metastasis, providing an experimental basis and theoretical support for future clinical tumor immunotherapeutic strategies.

## 4. Materials and Methods

### 4.1. Materials

Doxorubicin DOX and Calcein AM/PI Living and Dead Cell Stain Kit (100 T) were purchased from Dalian Meilun Biotechnology, Co., Ltd (Dalian, China). Sodium hyaluronate (HA, 40 kDa) was obtained from the Shandong Freda Biochem Co., Ltd. (Shandong, China). PEI (1.8 kDa) and methyl thiazolyl tetrazolium (MTT) were purchased from Sigma-Aldrich (America). RGD (liner) peptides were provided by Chinapeptides Co. Ltd. (Shanghai, China). Annexin V-FITC/PI Apoptosis Kit (100 T) was obtained from Jiangsu Kaiji Biotechnology Co., Ltd. (Jiangsu, China). QIAGEN Plasmid Extraction Kit (5 T, Qiagen Bioinformatics, Germany), CBA Multifactor Assay Kit (BD Biosciences, USA), Ptpn2 monoclonal antibody (Catalog No. 58935S), Calreticulin (CRT) monoclonal antibody (Catalog No. 12238S), and HMGB1 monoclonal antibody (Catalog No. 6893S) were purchased from Cell Signalling Technology, USA. CD4-BV510 monoclonal antibody (740122), CD8-percp-Cy5.5 (Catalog No. 553031), CD3-APC-Cy7 (Catalog No. 560590), and CD45-FITC (Catalog No. 557235) were purchased from BD Biosciences, USA; Mouse CD11B-BV605 monoclonal antibody (Catalog No. 101257), CD11C-BV711 (Catalog No. 117349), CD11c-PE (Catalog No. 12011482), CD86-APC (Catalog No. 17086282), and CD80-FITC (Catalog No. 11080942) were purchased from Biolegend, USA. Dimethyl sulfoxide anhydrous ethanol, isopropyl alcohol, ethylene glycol, chloroform, and other reagents were purchased from Chengdu Kelon Chemical Reagent Company.

### 4.2. Cells and Animals

The murine melanoma cell line, B16F10, and the macrophage cell line, RAW264.7, were purchased from the American Type Culture Collection and were cultured with RPMI 1640 or DMEM supplement with 10% FBS in incubators at 37°C containing 5% CO_2_ atmosphere.

Female C57BL/6 mice, 6-8 weeks of age, were purchased from Beijing Huafucang Biotechnology Co., Ltd (Beijing, China). All animal experiments were approved by the animal ethics committee of Sichuan University.

### 4.3. shRNA-Ptpn2

The plasmid pGPU6/GFP/Neo was used to construct the shRNA of Ptpn2 containing the green fluorescent protein- (GFP-) labeled fragment. The shRNA-Ptpn2 (Forward: 5′-CACAAAGAAGTTACATCTT-3′, Reverse: 3′-AAGATGTAACTTCTTTGTG-5′) was constructed by Gemma Gene-RNAi Professional Co., Ltd (Shanghai, China).

### 4.4. Synthesis of HA-DOX

HA (0.4 g, 1 mM) was dissolved in 10 mL of ddH_2_O, subsequently, 5 mL of ddH_2_O containing NaIO_4_ (0.053 g, 0.25 mM) was added. The solution was continuously stirred in the dark for 12 h, followed by the addition of ethylene glycol and stirring for another 0.5 h to terminate the reaction. The resulting solution was purified by dialysis (Mn = 1000 Da) to remove NaIO_4_, followed by lyophilization to obtain the OHA. Next, the 200 mg OHA and 10 mg DOX hydrochloride were added to 30 mL of ultrasound-degassed ddH_2_O. The reaction was conducted at 50°C for 48 h without light under N_2_. The red spongy product, HD, was obtained by freeze-drying, and the grafting rate of DOX was determined via UV-vis. The product was stored in the dark until further use. OHA and HD were further characterized using ^1^H-NMR and FTIR.

### 4.5. Preparation and Characterization of M1HD@RPR

#### 4.5.1. M1 Membrane Preparation

M1 macrophages were prepared by stimulating RAW264.7 cells with 100 ng mL^–1^ lipopolysaccharide (LPS) and 10 ng.mL^–1^ IFN-*γ* for 24 h. Thereafter, the cells were collected via centrifugation (1500 rpm, 3 min) and suspended with precooled Tris-magnesium salt buffer (TM buffer, pH 7.4, 0.01 M Tris, and 0.001 M MgCl_2_) at a concentration of 2 × 10^7^ cells/mL^–1^ and extruded through a microextruder (Avanti, LF-1, USA) without polycarbonate filter at least 20 times to destroy the cells [32]. Next, the cell membranes were extracted using the sucrose gradient centrifugation method. Briefly, 1 M sucrose solution was used to dilute the TM buffer to a final concentration of 0.25 M and was centrifuged (2000 g, 4°C for 10 min). The supernatant was collected and centrifuged (4000 g, 4°C for 30 min). Next, the precipitates were suspended and washed with a TM buffer solution of 0.25 M sucrose, centrifuged (6000 g, 4°C for 30 min) to collect the cell membranes, and subsequently subjected to ultrasound at 100 W for 2 min. The protein content of extracted membranes from 1 × 10^8^ cells was quantified via BCA assay and found to be 0.17 mg.

#### 4.5.2. M1HD@RPR Preparation

M1HD@RPR was prepared via the electrostatic adsorption method. In brief, 5 *μ*g shRNA-Ptpn2 plasmid was mixed with 25 *μ*g iRGD for 5 min to form a blend of iRGD and shRNA-Ptpn2 (iRGD-shRNA-Ptpn2, RR). Next, 125 *μ*g PEI was added and mixed for another 5 min, resulting in a complex of PEI and RP (iRGD-PEI-shRNA-Ptpn2, RPR). After mixing for 5 min, HA-DOX containing 50 *μ*g DOX was added to obtain HD@RPR (HA-DOX@iRGD-PEI-shRNA-Ptpn2). Finally, the HD@RPR were coated with M1 macrophage membranes (5 *μ*g shRNA-Ptpn2 added to 0.034 mg M1 macrophage membranes) to prepare M1HD@RPR by extrusion through a polycarbonate membrane with pore sizes of 400 nm and 200 nm. Meanwhile, PEI, iRGD, and OHA in the same amounts as M1HD@RPR were used to prepare blank nanoparticles (Blank NPs) by self-assembly.

The particle size, polydispersion index, and zeta potential of HD@RPR and M1HD@RPR nanoparticles were measured by DLS (Malvern, Zetasizer NanoZS ZEN3600, UK). The morphology of HD@RPR and M1HD@RPR was observed using a transmission electron microscope (Tecnai G2 F20 S-TWIN, USA).

To confirm that the M1 macrophage membranes coated the HD@RPR nanoparticles, SDS-PAGE assay was used to analyze the protein profiles. The M1HD@RPR nanoparticles were centrifuged (12,000 rpm for 30 min), and the membrane proteins were extracted from the M1 macrophage membranes and M1HD@RPR nanoparticles with RIPA lysis buffer and further measured using an electrophoresis assay. HD@RPR nanoparticles without M1 macrophage membranes were used as the control.

Gel block assay was performed to determine the protective effect of the nanodrug delivery system against shRNA-Ptpn2. RP, shRNA-Ptpn2, RPR, and HD@RPR were diluted with distilled water and adjusted to an equal content of shRNA-Ptpn2 (200 ng shRNA-Ptpn2 per well) and then loaded into 1.0% agarose gel in TAE buffer containing 0.6 *μ*g mL^–1^ ethidium bromide. Electrophoresis was performed at a constant voltage of 110 V for 40 min. Thereafter, the gel imaging system was used to collect and analyze the DNA retardation.

### 4.6. DOX Release

The pH-sensitive release of DOX was investigated using the dialysis method. Briefly, M1HD@RPR or HA-DOX solution (1 mL) containing 1.0 mg DOX was placed in a dialysis bag with an 8-14 kDa cut off. This was followed by immersion in 15 mL of PBS buffer at various pH (pH 5.7, 6.8, and 7.4) at 37°C with continuous oscillation at 100 rpm. All dialysis media was replaced with fresh PBS at pre-determined time intervals (0.5 h, 1 h, 2 h, 4 h, 8 h, 12 h, 1 d, 2 d, 4 d, 12 d, and 24 d). The DOX content was determined via UV-vis at 482 nm. The cumulative release rate was calculated, and the release curve was drawn.

### 4.7. In Vitro Cellular Uptake and Permeability

To investigate the uptake rate of nanoparticles by B16F10 cells, B16F10 cells were seeded in 24-well plates at a rate of 5 × 10^4^ cells per well. After 24 h, the medium was discarded and replaced with fresh serum-free medium containing free DOX, HD@RPR, or M1HD@RPR in which the final concentration of DOX was 5 *μ*g.mL^–1^. After 0.5 h, 1 h, and 2 h, cells were washed and analyzed via flow cytometry (ACEA, USA).

The intracellular distribution of the Free DOX, HD@RPR, and M1HD@RPR in B16F10 cells was examined via confocal laser scanning microscope (CLSM) (ZEISS, LSM 880, Germany). In brief, B16F10 cells were seeded in a 24-well plate over glass coverslips (8 × 10^4^ cells/well). The nanoparticles containing DOX with a final concentration of 5 *μ*g.mL^–1^ were added to each well. After 0.5 h, 1 h, and 2 h, cells were washed and subsequently fixed with 4% paraformaldehyde for 15 min. After being washed with PBS again, the cells were permeabilized with 0.2% Triton X-100 for 10 min and blocked with 3% BSA at 25°C for 10 min. Thereafter, the cells were incubated with FITC-phalloidin for 60 min to localize actin filaments. Finally, the nuclei were labeled with 1 *μ*g mL^–1^ DAPI for 10 min and photographed under a Zeiss microscope.

To investigate the uptake efficiency of nanoparticles by M0 and M1 macrophages, an M1 macrophage cellular uptake assay was performed, for which RAW264.7 cells (M0 macrophages) were polarized into M1 macrophages by the method mentioned above. Next, the M0 and M1 macrophages were seeded in a 24-well plate over glass coverslips at a concentration of 8 × 10^4^ cells per well. After 24 h, Free DOX, HD@RPR, or M1HD@RPR were added for 2 h. Thereafter, cells were washed and further stained with DAPI for 10 min to stain the nucleus and then observed with CLSM.

Finally, 3D tumor spheres were established to investigate the effect of nanoparticles on tumor penetration. In brief, 1 mL of 2% low melting point agarose was added into a 6-well plate. Next, B16F10 cells were seeded at a concentration of 2 × 10^5^ cells/well, and 5 days later, the tumor spheroids were treated with free DOX, HD@RPR, or M1HD@RPR (final DOX concentration, 5 *μ*g mL^–1^). After 2 h, tumor spheroids were centrifuged (100 rpm, 3 min) twice with PBS and transferred to confocal petri dishes for laser confocal imaging.

### 4.8. Ptpn2 Gene Silencing In Vitro

B16F10 cells (2 × 10^5^ cells/well) were seeded into a 6-well cell culture plate. After 24 h, M1HD@RPR, naked shRNA-Ptpn2, and Lipofectamine 3000, with 1 *μ*g shRNA-Ptpn2 per well were added. Meanwhile, blank medium was added as a control group. Cells were collected after 24 h for western blotting.

### 4.9. Cellular ICD Response Assay

To investigate the ICD effect induced by DOX, the expression of Calreticulin (CRT) and High Mobility Group Box 1 (HMGB1) protein in cells was evaluated by immunofluorescence staining. Briefly, B16F10 cells were inoculated in a 24-well plate containing a glass slide and incubated with PBS, Free DOX, HD@RPR, and M1HD@RPR for 2 h. Thereafter, cells were washed and stained with anti-CRT or anti-HMGB1 primary antibody at 25°C for 2 h followed by washing and incubation with FITC-labeled fluorescent secondary antibodies for 2 hours. Finally, the nuclei were stained with DAPI and imaged under a confocal microscope (Zeiss, Germany).

### 4.10. Apoptosis and Calcein-AM/PI Staining

B16F10 cells (2 × 10^5^ per well) were seeded into 6-well plates and incubated at 37°C for 24 h. The media was then replaced with 2 mL complete medium containing different compositions: PBS, Blank NPs, M1 membranes treated alone (M1), nanoparticle with shRNA alone (OHA@RPR), free DOX, HD@RPR, or M1HD@RPR. The shRNA-Ptpn2 content was 0.5 *μ*g per well, and the final DOX concentration was 2.5 *μ*g.mL^–1^. After 16 h, the supernatant medium of each group was collected. Thereafter, cells were trypsinized and stained with the Annexin V-FITC Apoptosis detection kit. Flow cytometry (ACEA, USA) was used to detect FITC and propidium iodide (PI) fluorescence.

For the calcein-AM/PI staining assay, the concentration of each composition in the apoptosis test was doubled. After incubation for 16 h, cells were stained with calcein-AM/PI and imaged using an inverted fluorescent microscope (Olympus, Japan).

### 4.11. Antitumor Immunity Study In Vitro

To simulate the effect of nanoparticles on bone marrow-derived dendritic cell (BMDC) maturation or M1 macrophage polarization in a tumor microenvironment, B16F10 cells were inoculated into a 24-well plate, after which PBS, Blank NPs, M1, OHA@RPR, Free DOX, HD@RPR, and M1HD@RPR were added and incubated for 24 h. The cell supernatant was then collected from each well.

#### 4.11.1. BMDC Maturation Assay

C57BL/6 mice were sacrificed followed by excision of the distal tibia and femur. The bone marrow was rinsed with RPMI 1640 medium. After the red blood cells (RBCs) were lysed with erythrocyte lysate, the remaining cells were collected by centrifugation (500 g for 5 min). Thereafter, the cells were cultured in complete RPMI 1640 medium containing a final concentration of 20 ng.mL^–1^ GM-CSF and 10 ng.mL^–1^ IL-4, with fresh medium replaced every other day. The BMDCs were generated after 6 days and were collected for seeding into a 24-well plate. After 24 h, the supernatant of the B16F10 cells, as prepared above, was diluted with complete medium and then used to replace the original BMDC medium. After another 24 h, BMDCs were stained with fluorescence antibodies (CD11c-PE, CD86-APC, and CD80-FITC). The maturation rate of BMDCs was analyzed by flow cytometry.

#### 4.11.2. M1 Macrophages Polarization Analysis

RAW264.7 cells were seeded into a 24-well plate for 24 h. Next, the original medium was replaced with the supernatant of B16F10 cells diluted by DMEM. After incubation for another 24 h, cells were stained with CD86-APC antibody. The M1-polarized macrophages were analyzed by flow cytometry.

Meanwhile, within the tumor microenvironment, a large proportion of the macrophages are M2-polarized [45]. Therefore, M0 cells were polarized into M2 using fresh complete medium supplemented with IL-4 and IL-10 (20 ng.mL^–1^) for 24 h. The supernatant of B16F10 cells was prepared, as described above, and then diluted 5 times with high glucose DMEM to replace the original M2 macrophage medium. After 24 h, M1 macrophages in each group were stained with F4/80-APC, CD86-PE, and CD206-PE/cy7, and their relative proportion was measured by flow cytometry.

Furthermore, the appearance of M1 polarized macrophages was observed by immuno-fluorescence staining. After cells were fixed with paraformaldehyde, the anti-CD86 primary antibody was added and incubated for 2 h at 25°C. Finally, the fluorescence secondary antibody labeled with FITC was added and incubated for another 2 h. The images were obtained and observed under a confocal microscope.

### 4.12. Tissue Distribution

Female C57BL/6 mice (16–18 g) were subcutaneously injected in the right flank with 2 × 10^5^ B16F10 cells in 100 *μ*L serum-free 1640 medium. Mice were randomly divided into three groups as the volume of tumors reached 300-500 mm^3^. Free DOX, HD@RPR, or M1HD@RPR were intravenously injected into mice at a dose of 2.5 mg/kg. At 1, 4, 12, and 24 h, the blood of each mouse was sampled, and the brain, heart, liver, lung, and kidney were dissected, weighed, triturated, and homogenized with saline at 4°C. The DOX was extracted using 100 *μ*L methanol, 400 *μ*L chloroform, and 50 *μ*L inner standard (10 *μ*g.mL^–1^ DOX in methanol). After vortexing and centrifugation (12,000 rpm, 10 min), the chloroform phase was collected, transferred to a new tube, and dried under nitrogen protection at 40°C. Samples at the bottom of the tube were suspended with high-performance liquid chromatography (HPLC) methanol. After centrifugation, the supernatant was collected, and DOX was detected via HPLC.

To directly observe the tumor accumulation following DOX administration, 24 h post-DOX administration, a portion of the fresh tumors were fixed in 4% paraformaldehyde. Next, 10 *μ*m thick tumor sections were cut, and the nucleus was stained with DAPI. Finally, imaging was done by CLSM.

### 4.13. Antitumor Therapeutic Effects In Vivo

For primary tumor treatment, B16F10-bearing C57BL/6 mice were randomly divided into seven groups (*n* = 5). After seven days, the tumor volume reached approximately 100 mm^3^, at which point the mice were treated intravenously with PBS, Blank NPs, M1, OHA@RPR, Free DOX, HD@RPR, and M1HD@RPR every two days. A total of three injections were administered to each mouse. The doses of DOX and the shRNA-Ptpn2 were 2.5 mg.kg^–1^ and 5 *μ*g per mouse, respectively. Tumor size and body weight were measured every two days (tumor volume = length × width^2^ × 0.5). After the final measurement, mice were sacrificed at day 15, and the heart, liver, spleen, lung, kidney, and tumor were collected and fixed in 4% paraformaldehyde (PFA) for further H&E staining. In addition, parts of the fixed tumor tissues were further immunohistochemically (IHC) stained (TUNEL, Ki67, staining).

To evaluate the chemo-immunotherapeutic-induced antitumor effect, C57BL/6 mice were divided randomly into seven groups (*n* = 4) and challenged intravenously with 2 × 10^5^ B16F10 cells. The following day, mice were treated intravenously with PBS, Blank NPs, M1, OHA@RPR, Free DOX, HD@RPR, or M1HD@RPR every two days, and a total of three injections were given. The doses of DOX and the shRNA-Ptpn2 were 2.5 mg.kg^–1^ and 5 *μ*g per mouse, respectively. After 10 days of treatment, the mice in the control group presented with rickets and slow movement. The mice were euthanized, and the lung tissues were taken out, photographed, further fixed with paraformaldehyde, and embedded in paraffin for H&E staining.

### 4.14. Ptpn2 Gene Silencing In Vivo

To investigate the silencing effect of nanoparticles *in vivo*, fresh tumor tissues from the mice in each group were collected after treatment, frozen in liquid nitrogen, and ground into powder in a mortar. The total RNA was isolated using the Trizol lysate method and reversed transcribed to cDNA using the PrimeScript™ RT reagent kit (Takara, Japan). Next, qPCR (quantitative real-time PCR) amplification was performed using the CFX96 Real-Time PCR Detection System (Bio-Rad). The primers are shown in Supplementary Table 1. Tumors were fixed with 4% PFA for the Ptpn2 IHC assay.

### 4.15. In Vivo ICD Effect Assay

To investigate the ICD effect induced by DOX *in vivo*, the mRNA expression levels of CRT and HMGB1 in tumor tissues were detected, and qPCR was performed using similar methods to those described above. The primers are also shown in Supplementary Table 1. In addition, a portion of the tumors was fixed with 4% PFA for CRT IF staining.

### 4.16. Antitumor Immunity Study In Vivo

To analyze the immunotherapeutic effect of shRNA-Ptpn2 on primary tumors, mice were sacrificed following the last measurement and blood collection. RBCs were removed from the blood samples via RBC lysis buffer. Additionally, the fresh tumor tissues were digested into single cells by collagenase IV and DNase I. Thereafter, the single immune-associated cells from tumors and the whole blood were obtained and stained with different fluorescent antibodies (CD45-FITC, CD3-APC-cy7, CD4-bV510, CD8-Percp cy5.5, CD86-APC, CD11b-BV605, CD11c-BV711, and F4/80-PE) according to the manufacturer's instructions. The levels of cytokines IFN-*γ*, TNF-*α*, and IL-6 in the serum were measured by the corresponding CBA cytokine quantitative kit, and simultaneously, the mRNA expression levels of IFN-*γ*, TNF-a, and IL-6 in tumor tissues were detected by qPCR using similar methods to those described above; all primers are shown in Supplementary Table 1.

### 4.17. Safety Evaluation

To evaluate the safety of nanoparticles *in vivo*, routine blood and biochemical analyses were performed on mice. In brief, healthy C57BL/6 mice were intravenously injected with PBS, Blank NPS, M1, OHA@RPR, Free DOX, HD@RPR,M1HD@RPR(4 mice in each group) on the 1st, 3rd, and 5th day of the study. On day 10, whole blood was collected to determine the levels of white blood cells, RBCs, platelets, hematocrit, and hemoglobin. Meanwhile, serum was collected to determine the levels of alanine transaminase (ALT), urea, creatinine (CREA), and aspartate transaminase (AST).

### 4.18. Statistical Analysis

Statistical analysis was performed using GraphPad Prism 5 software (GraphPad Software, Inc., La Jolla, CA), and the results were expressed as mean ± standard deviation. One-way analysis of variance (ANOVA) was used for statistical analysis, and the *t*-test was used for comparison between the two groups. In this study, significant differences between groups were indicated ^∗^*P* < 0.05, ^∗∗^*P* < 0.01, ^∗∗∗^*P* < 0.001, and ^∗∗∗∗^*P* < 0.0001.

## Data Availability

All data used to support the findings in the paper and supplementary materials are available from the corresponding authors upon reasonable request.

## Supplementary Materials

Supplementary Materials Supplementary Table 1: primers used for mice qPCR. Figure S1: scheme of the synthesis of HA-DOX. Figure S2: physicochemical characterization of HA-DOX. ^1^H-NMR and FTIR spectra of (a) HA; (b) OHA; (c) DOX; (d) HA-DOX. Figure S3: Calcein-AM/PI detected the death of B16F10 cells induced by each group (200 *μ*m). Figure S4: representative flow cytometry plots of CD11c^+^CD80^+^ and CD11c^+^CD86^+^ ratio. Figure S5: flow cytometry results of M1 macrophages polarization by nanoparticles. Figure S6: H&E-stained images of major organs treated with each formulation (scale = 100 *μ*m). Figure S7: representative flow cytometry plots of CD8^+^ T cells proportion in the tumor. Figure S8: representative flow cytometry plots of CD11c^+^CD86^+^ cells proportion in the tumor. Figure S9: representative flow cytometry plots of F4/80^+^ CD86^+^ cells proportion in the tumor. Figure S10: representative flow cytometry plots of CD8^+^ T cells proportion in the blood. Figure S11: representative flow cytometry plots of CD11c^+^CD86^+^ cells proportion in the blood. Figure S12: representative flow cytometry plots of F4/80^+^ CD86^+^ cell proportion in the blood. Figure S13: mRNA expression of IL-6, TNF-*α*, and IFN-*γ* detected via qPCR after treatment with different groups. Figure S14: cytokine levels in sera after treatment with different nanoformulations. The cytokine levels were measured using a mouse inflammation kit via flow cytometry. Figure S15: blood routine analysis and blood biochemical analysis of mice in different groups after treatment (blood routine: red; blood biochemistry: blue).
