# Supplementary material for: Macrophage Membrane-Camouflaged shRNA and Doxorubicin: A pH-Dependent Release System for Melanoma Chemo-Immunotherapy
Source: Research (Wash D C). 2022 Feb 8;2022:9768687. doi: 10.34133/2022/9768687 (PMC8851070; doi:10.34133/2022/9768687)
Supplement: Supplementary Materials — Supplementary Table 1: primers used for mice qPCR. Figure S1: scheme of the synthesis of HA-DOX. Figure S2: physicochemical characterization of HA-DOX. 1H-NMR and FTIR spectra of (a) HA; (b) OHA; (c) DOX; (d) HA-DOX. Figure S3: Calcein-AM/PI detected the death of B16F10 cells induced by each group (200 μm). Figure S4: representative flow cytometry plots of CD11c+CD80+ and CD11c+CD86+ ratio. Figure S5: flow cytometry results of M1 macrophages polarization by nanoparticles. Figure S6: H&E-stained images of major organs treated with each formulation (scale = 100 μm). Figure S7: representative flow cytometry plots of CD8+ T cells proportion in the tumor. Figure S8: representative flow cytometry plots of CD11c+CD86+ cells proportion in the tumor. Figure S9: representative flow cytometry plots of F4/80+ CD86+ cells proportion in the tumor. Figure S10: representative flow cytometry plots of CD8+ T cells proportion in the blood. Figure S11: representative flow cytometry plots of CD11c+CD86+ cells proportion in the blood. Figure S12: representative flow cytometry plots of F4/80+ CD86+ cell proportion in the blood. Figure S13: mRNA expression of IL-6, TNF-α, and IFN-γ detected via qPCR after treatment with different groups. Figure S14: cytokine levels in sera after treatment with different nanoformulations. The cytokine levels were measured using a mouse inflammation kit via flow cytometry. Figure S15: blood routine analysis and blood biochemical analysis of mice in different groups after treatment (blood routine: red; blood biochemistry: blue). [file 9768687.f1.docx]

Supplementary Materials for **Research**

**Macrophage membrane-camouflaged shRNA and doxorubicin: A pH-dependent release system for melanoma chemo-immunotherapy**

**Chengli Yang,^1,2^ Yang Ming,^1^ Kai Zhou,^1^ Ying Hao,^1^ Danrong Hu,^1^ Bingyang Chu,^1^ Xinlong He,^1^ Yun Yang,^1^ ZhiyongQian*^1^**

*^1^State Key Laboratory of Biotherapy and Cancer Center, West China Hospital, Sichuan University, and Collaborative Innovation Center of Biotherapy, Chengdu, Sichuan 610041, P. R. China.*

*^2^Department of Pharmacy,The Affiliated Hospital of Guizhou Medical University, Gui Yang, Gui Zhou 550000, P. R. China.*

*E-mail:* [*anderson-qian@163.com*](mailto:anderson-qian@163.com)*.*

**Supplementary Table 1**. Primers used for mice qPCR

| Gene | Forward (5ʹ–3ʹ) | Reverse (5ʹ–3ʹ) |
| --- | --- | --- |
| Ptpn2 | CCAACTCAGATTCTCCTACA | CTCTCCTCCACAGTATCCT |
| CRT | GCCAGACAACACCTATGAG | CCACTCTCCATCCATCTCT |
| HMGB1 | GCCTTCTTCTTGTTCTGTTC | ATCCTCCTCATCATCTTCCT |
| IL-6 | TCCATCCAGTTGCCTTCT | TAAGCCTCCGACTTGTGA |
| TNF-α | GTGGAACTGGCAGAAGAG | TAGACAGAAGAGCGTGGT |
| IFN-γ | GTGGCATAGATGTGGAAGA | GTGTGATTCAATGACGCTTA |


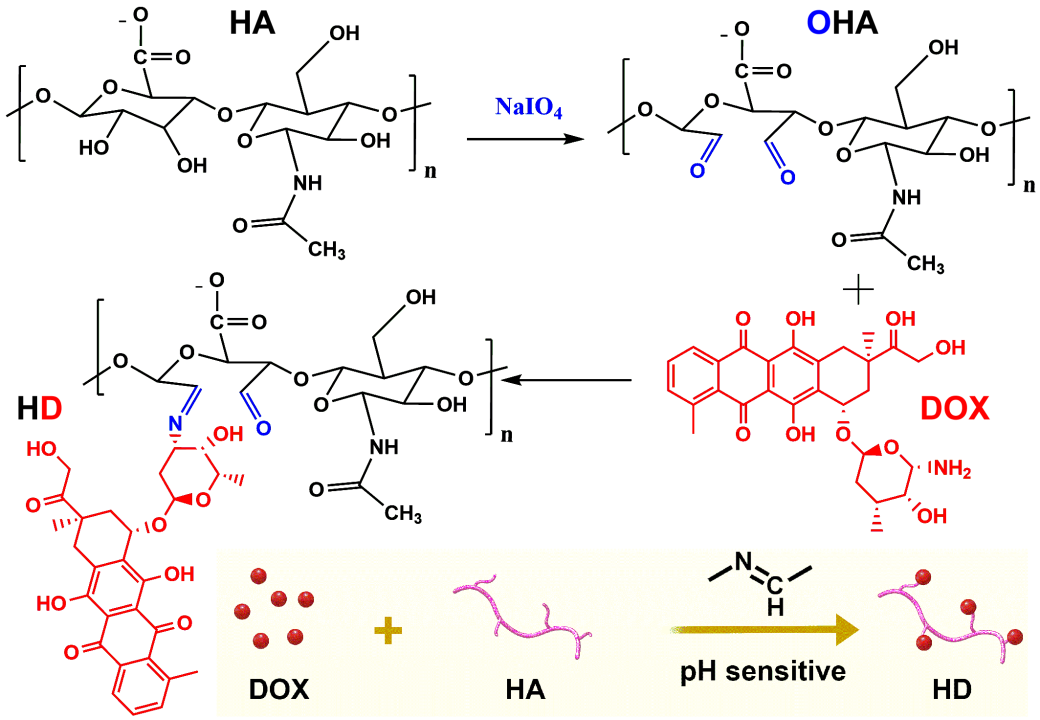


**Figure S1.** Scheme of the synthesis of HA-DOX


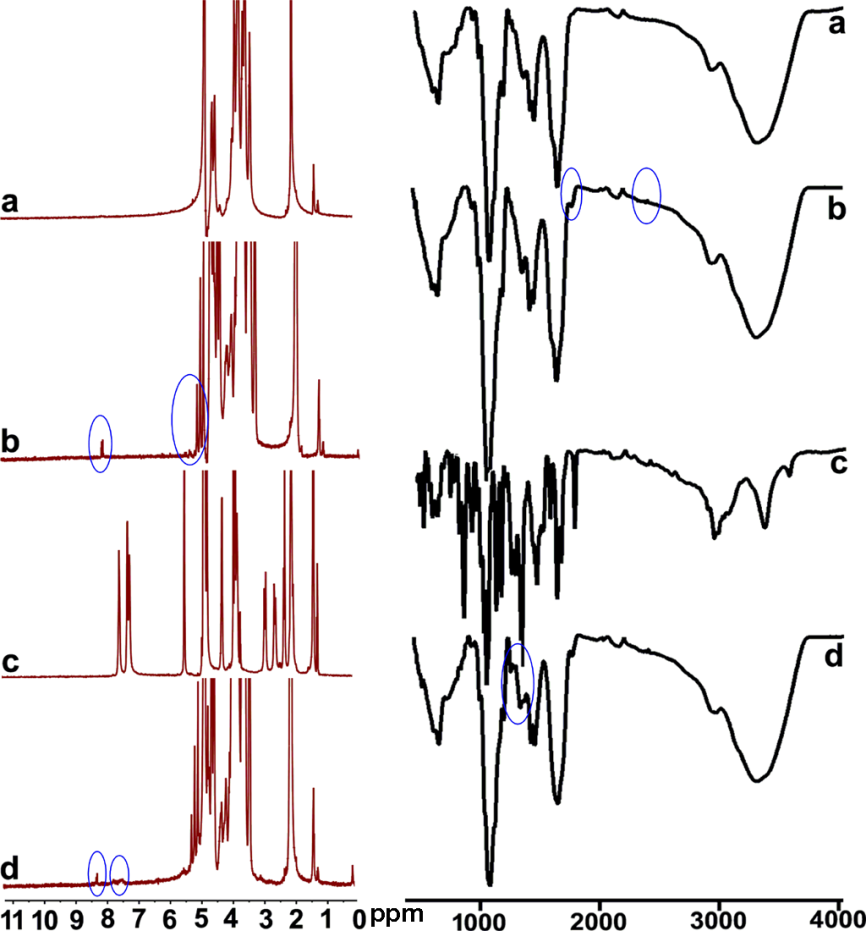


**Figure S2.** Physicochemical characterization of HA-DOX. ^1^H-NMR and FTIR spectra of a, HA; b, OHA; c, DOX; d, HA-DOX.

**
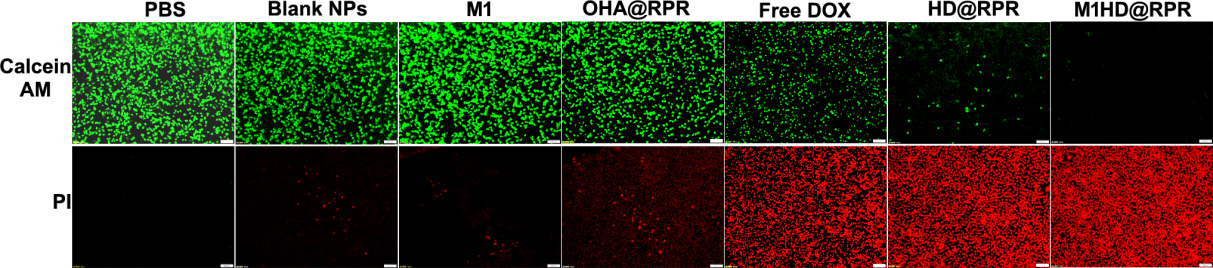
**

**Figure S3.** Calcein-AM /PI detected the death of B16F10 cells induced by each group (200 μm).


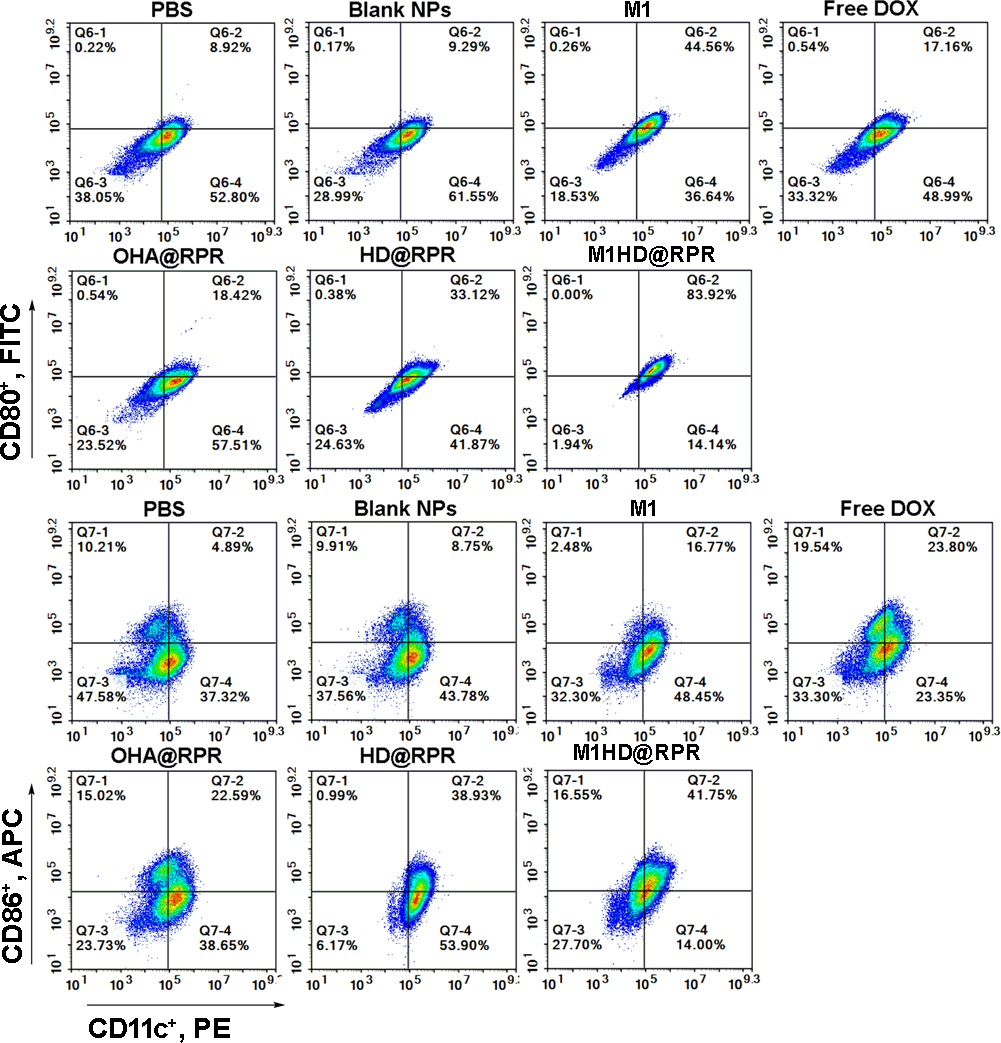


**Figure S4.** Representative flow cytometry plots of CD11c^+^ CD80^+^ and CD11c^+^ CD86^+^ ratio.


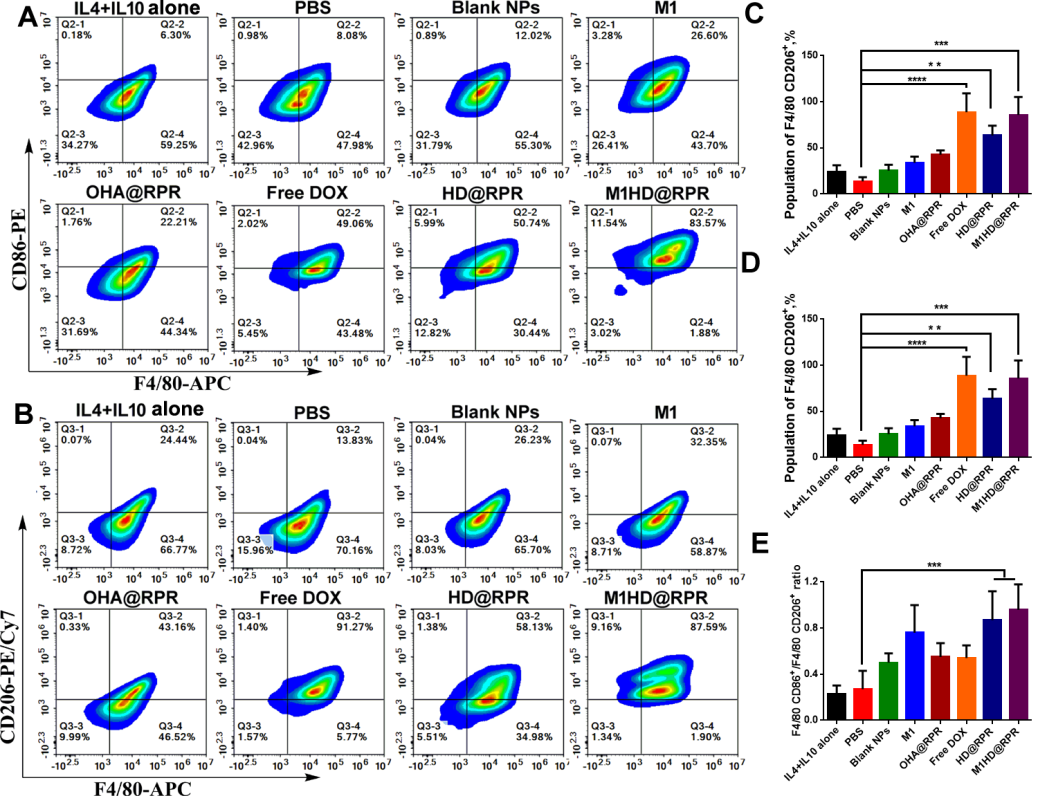


**Figure S5.** Flow cytometry results of M1 macrophages polarization by nanoparticles. (A) and (C) Representative flow cytometry plots of F4/80^+^ CD86^+^ cells proportion. (B) and (D) Representative flow cytometry plots of F4/80^+^ CD206^+^ cells proportion.(E) M1/M2 ratio result.


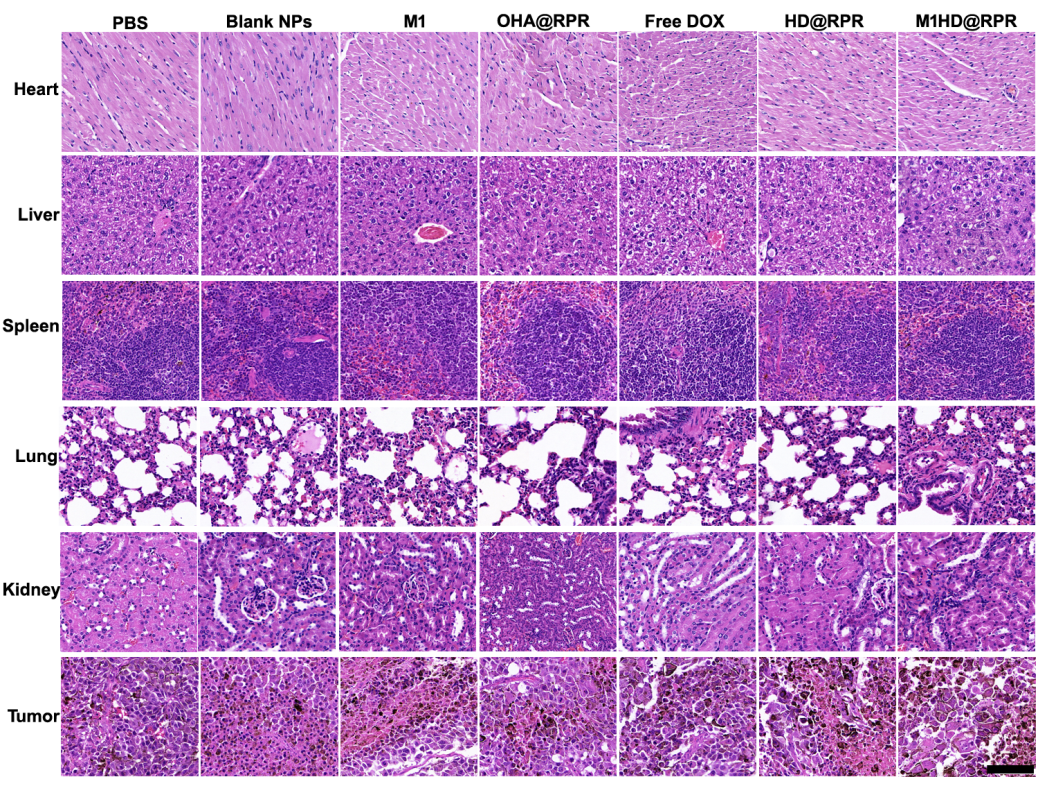


**Figure S6.** H&E-stained images of major organs treated with each formulation (scale =100 μm).


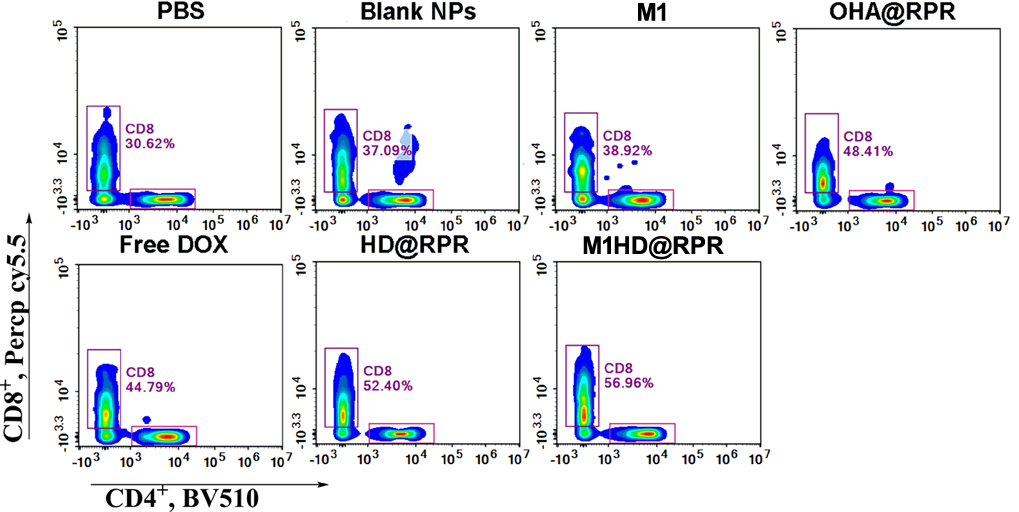


**Figure S7.** Representative flow cytometry plots of CD8^+^ T cells proportion in the tumor.


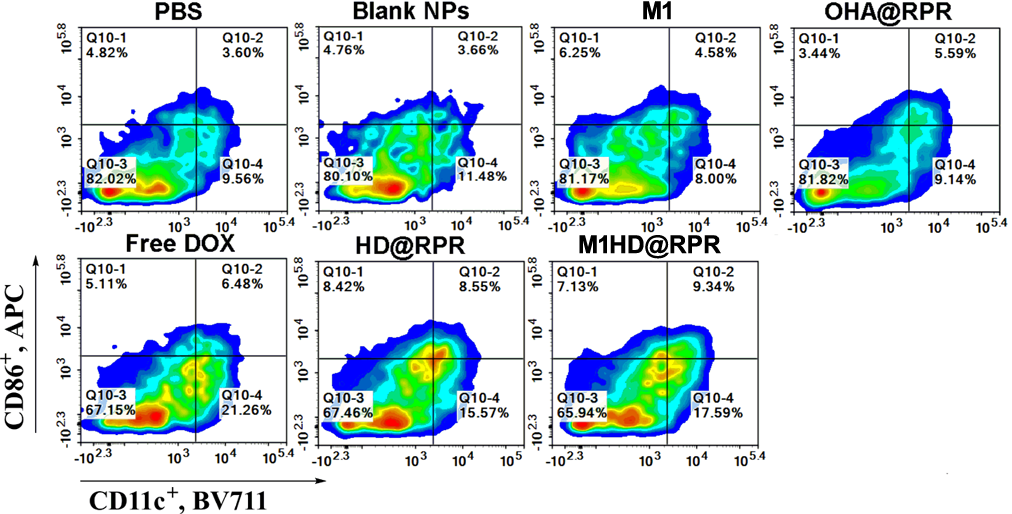


**Figure S8.** Representative flow cytometry plots of CD11c^+^CD86^+^ cells proportion in the tumor.


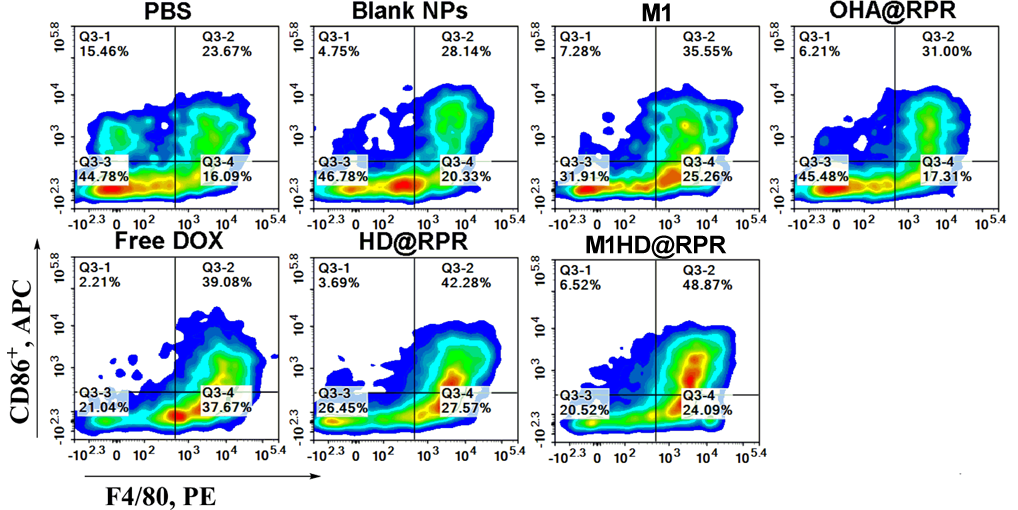


**Figure S9.** Representative flow cytometry plots of F4/80^+^ CD86^+^ cells proportion in the tumor.


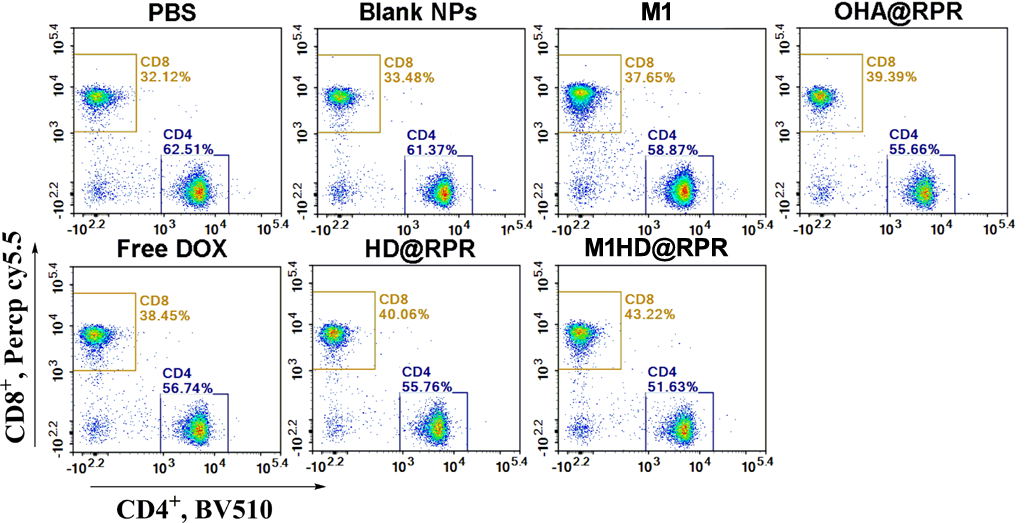


**Figure S10.** Representative flow cytometry plots of CD8^+^ T cells proportion in the blood.


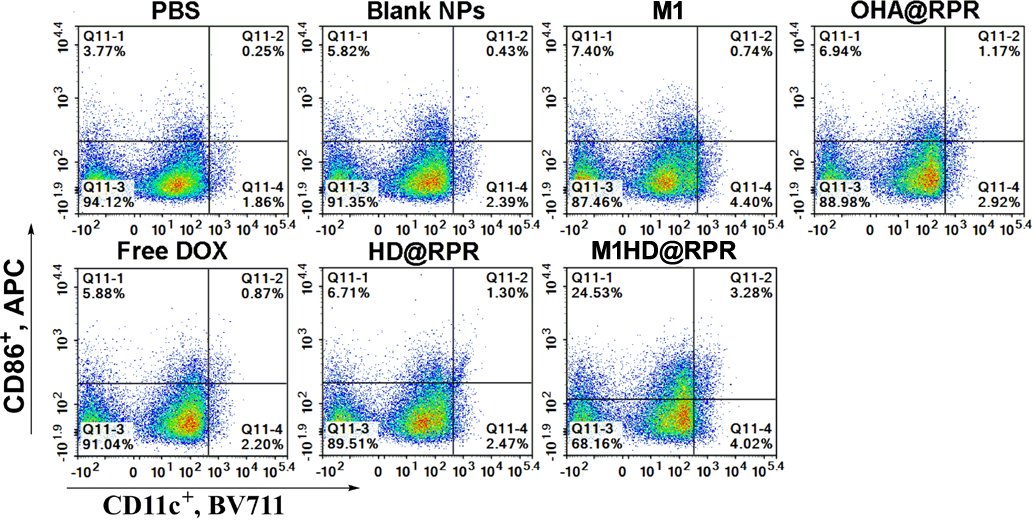
.

**Figure S11.** Representative flow cytometry plots of CD11c^+^CD86^+^ cells proportion in the blood.


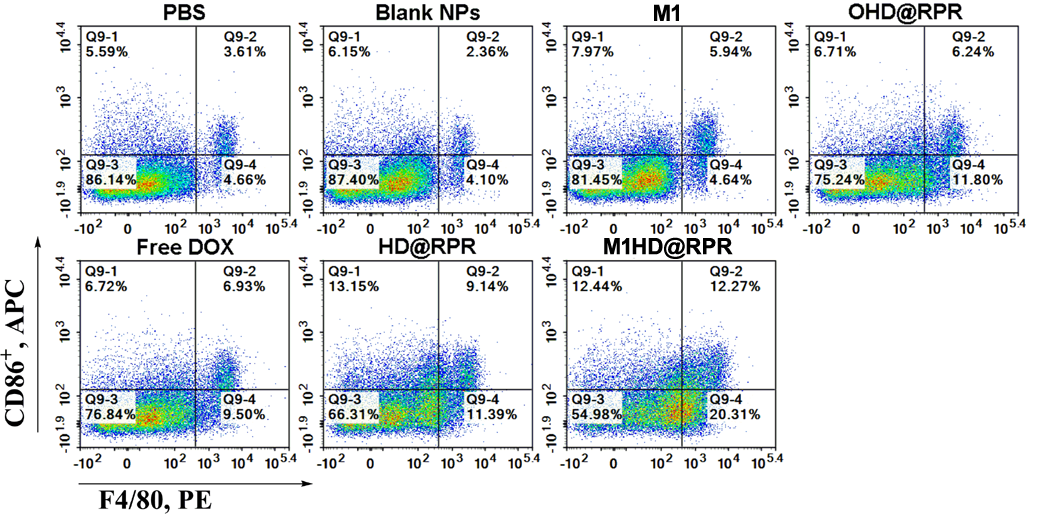


**Figure S12.** Representative flow cytometry plots of F4/80+ CD86+ cells proportion in the blood.


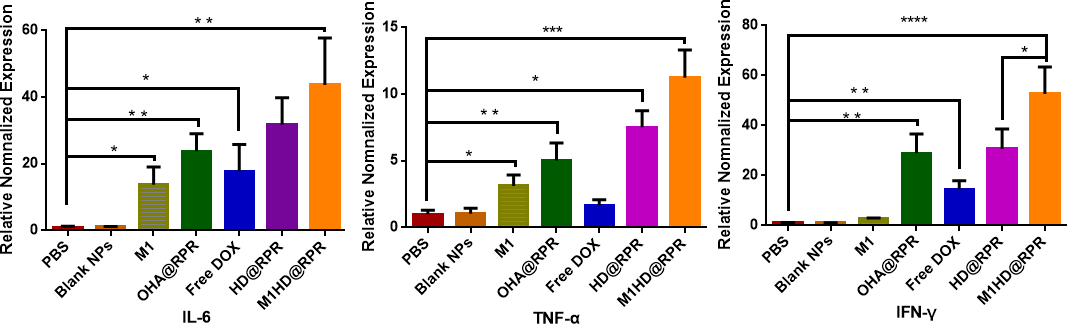


**Figure S13**. mRNA expression of IL-6, TNF-α, and IFN-γ detected via qPCR after treatment with different groups.
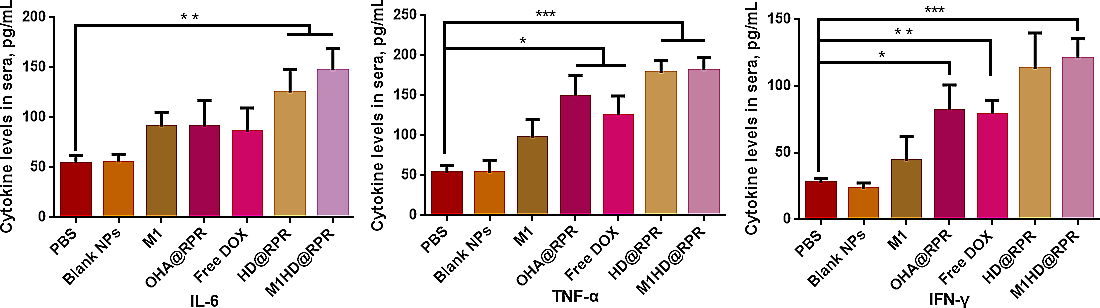


**Figure S14.** Cytokine levels in sera after treatment with different nanoformulations. The cytokine levels were measured using a mouse inflammation kit via flow cytometry.


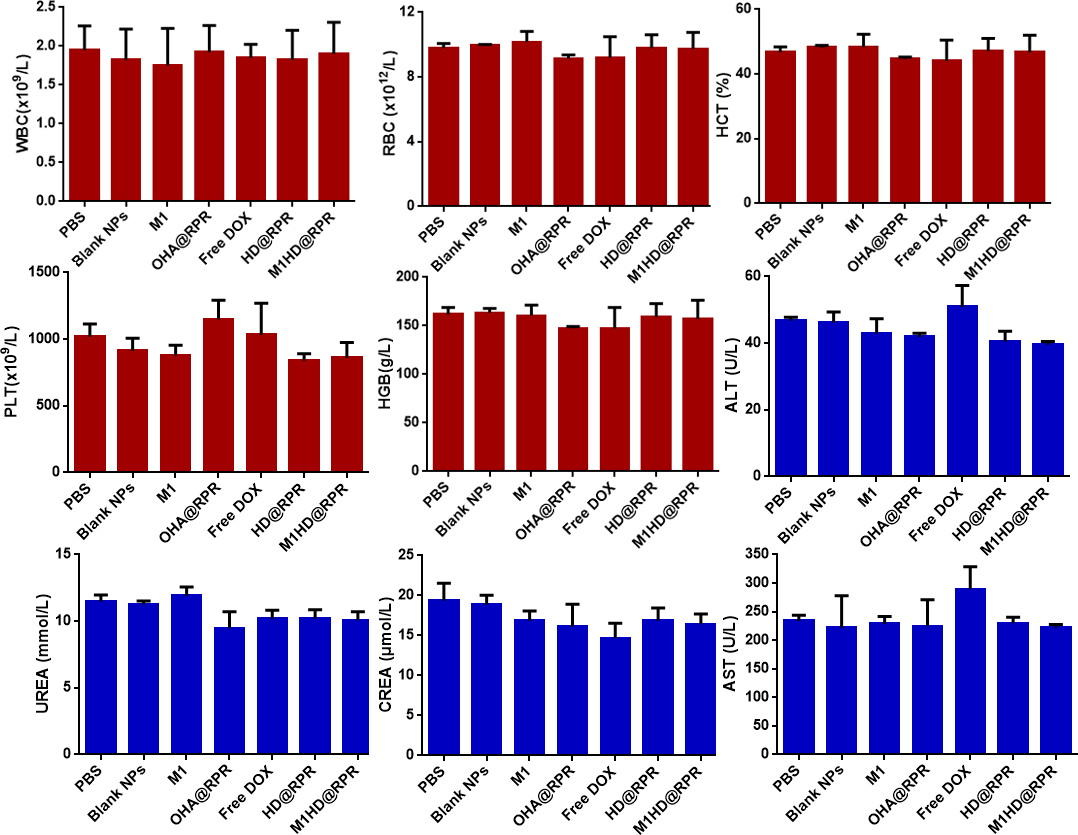


**Figure S15.** Blood routine analysis and blood biochemical analysis of mice in different groups after treatment (Blood routine: red; Blood biochemistry: blue).
